# Socioeconomic Disparities in Cancer Treatment, Service Utilization and Catastrophic Health Expenditure in China: A Cross-Sectional Analysis

**DOI:** 10.3390/ijerph17041327

**Published:** 2020-02-19

**Authors:** Yang Zhao, Lin Zhang, Yu Fu, Minyu Wang, Luwen Zhang

**Affiliations:** 1 Melbourne School of Population and Global Health, The University of Melbourne, Melbourne, Victoria 3010, Australia; zhaoyang001@hsc.pku.edu.cn (Y.Z.); yff1@student.unimelb.edu.au (Y.F.); 2 WHO Collaborating Centre on Implementation Research for Prevention & Control of NCDs, Melbourne, Victoria 3010, Australia; 3 Centre for Epidemiology and Biostatistics, Melbourne School of Population and Global Health, The University of Melbourne, Melbourne, Victoria 3010, Australia; tony1982110@gmail.com; 4 The University of Melbourne Centre for Cancer Research, Victorian Comprehensive Cancer Centre, Melbourne, Victoria 3000, Australia; 5 Cancer Immunology Program, Peter MacCallum Cancer Centre, Melbourne, Victoria 3000 Australia; Minyu.WANG@petermac.org; 6 Sir Peter MacCallum Department of Oncology, The University of Melbourne, Parkville, Victoria 3052, Australia; 7 Centre for Cancer Immunotherapy, Peter Mac and VCCC Alliance, Melbourne, Victoria 3000, Australia; 8 School of Health Services Management, Southern Medical University, Guangzhou 500000, Guangdong, China

**Keywords:** socioeconomic disparities, cancer treatment, catastrophic health expenditure, financial burden, China

## Abstract

Background: This study aims (1) to assess socioeconomic disparities in healthcare use and catastrophic health expenditure (CHE) among cancer patients in China, which is defined as the point at which annual household health payments exceeded 40% of non-food household consumption expenditure, and (2) to examine the association of different treatments for cancers with health service utilization and CHE. Methods: We used nationally representative data from the China Health and Retirement Longitudinal Study in 2015 with 17,018 participants in which 381 with doctor-diagnosed cancer. The main treatments for cancer included the Chinese traditional medicine (TCM), western modern medicine (refers to taking western modern medications excluding TCM and other treatments for cancers), surgery, and radiation/chemotherapy. Concentration curve was used to assess economic-related disparities in healthcare and CHE. Multivariate regression models were used to examine the impact of the cancer treatment on health service use and incidence of CHE. Results: The main cancer treatments and health service use were more concentrated among the rich patients than among the poor patients in 2015. There was a positive association between the treatment of cancer and outpatient visit (Adjusted Odds Ratio (AOR) = 2.492, 95% CI = 1.506, 4.125), inpatient visit (AOR = 1.817, 95% CI = 1.098, 3.007), as well as CHE (AOR = 2.744, 95% CI = 1.578, 4.772). All cancer therapies except for medication treatments were associated with a higher incidence of CHE, particularly the surgery therapy (AOR = 6.05, 95% CI = 3.393, 27.866) in urban areas. Conclusion: Disparities in treatment and health service utilization among Chinese cancer patients was largely determined by financial capability. The current insurance schemes are insufficient to address these disparities. A comprehensive health insurance policy of expanding the current benefits packages and strengthening the Public Medical Assistance System, are essential for Chinese adults with cancer.

## 1. Introduction

Non-communicable diseases (NCDs) are major causes of health burdens, and cancer ranks as one of the leading causes in most countries in the world. Data from the Global Cancer Observatory (GCO) reported that 4.2 million new cancer cases were diagnosed and 2.9 million cancer deaths resulted in China in 2018, accounting for 23% and 30% worldwide [1]. Cancer relevant death accounts for one-fifth of the total deaths in China and brings a heavy financial burden to patients, their families, the whole healthcare and insurance system, and the society.

In 2009, China launched a new comprehensive health system reform, which focused on five areas: service delivery, essential medicines, public health service, social health insurance, and public hospital reform. The Chinese government has implemented a series of measures to ensure the provision of accessible and affordable care to cancer patients and protect their health and wellbeing since the 2009 health system reform [2,3]. Almost the entire Chinese population (more than 95% of total) has been covered by one of three social health insurance programs, including the Urban Employee Basic Medical Insurance (UEBMI) Scheme, the Urban Resident Basic Medical Insurance (URBMI) Scheme, and the New Rural Cooperative Medical Scheme. To improve fairness in health insurance coverage, by the end of 2015 the central government in China has announced the integration of URBMI and NRCMS into the new urban–rural resident medical insurance (URRMI) scheme. Universal health insurance coverage was established in China, and the level of financial protection and benefits packages for cancer patients increased fast with time [4,5]. Alongside, Critical Illness Medical Insurance, other supplementary insurances offer extra financial protections to cancer patients as another layer of safety net. The consolidation of the health delivery system and coordination of providers at three levels could provide coordinated diagnoses, treatment, and follow-up care to cancer patients [6]. We hypothesized that the expanded health insurance system would improve the financial protection for cancer patients in China. We used the catastrophic health expenditure (CHE), defined as the point at which annual household health payments exceeded 40% of non-food household costs, to measure the degree of financial risk protection.

Though measures have been taken nationally, it takes time to translate the investments into equitable healthcare utilization among sub-populations of different socioeconomic characteristics [7]. Several studies from China have well-documented associations between cancer and socioeconomic risk factors [8,9,10,11,12]. Most of the previous studies focus on the rural/urban, geographical/regional disparities [13,14,15,16], and sex disparities in cancer incidence and mortality [17,18], as well as disparities in treatment options [19]. In China, a few studies have examined the healthcare disparities among people with chronic diseases, such as hypertension, cardiovascular diseases, chronic obstructive pulmonary disease, and multimorbidity [20,21,22,23,24,25]. However, there is limited evidence of socioeconomic disparities in treatment, health service utilization, and financial protection among cancer patients following China’s health system reform in 2009 [26].

This study aims to: (1) examine socioeconomic disparities in the healthcare and of CHE among cancer patients in China, and (2) investigate the association of different treatments for cancers with health service utilization and CHE. We hope to disseminate our findings to the scientific community, policymakers, and healthcare providers.

## 2. Materials and Methods

### 2.1. Data Source

Data were extracted from the China Health and Retirement Longitudinal Study (CHARLS) Wave 2015. CHARLS is a nationally representative dataset designed to represent Chinese residents aged 45 and over to serve the needs of scientific research on the ageing population and their health needs [27]. CHARLS is designed based on the Health and Retirement Study (HRS) and other related ageing surveys. Its questionnaires cover the following domains: demographics, health status and functioning, healthcare and insurance, income and consumption, and a number of important biomarkers including height, weight, and blood pressure [27]. The dataset provides a comprehensive population-based source of information to study cancer care utilization, which includes the demographic, clinical, social, and economic status for persons with cancer, as well as the information on healthcare service and cost.

To ensure sample representativeness, the CHARLS sampled 150 counties/districts and 450 villages/urban communities, across 28 provinces, by using multi-stage stratified probability-proportionate-to-size (PPS) sampling. A total of 21,097 individuals were interviewed in 2015 (3rd wave). Ongoing follow-up surveys were conducted every two years [27]. After excluding cases with missing demographic information and/or treatment measurements, complete data were available for 17,018 individuals in 2015. Total of 381 participants were reported having doctor-diagnosed cancer.

### 2.2. Indicators

We identified four kinds of cancer treatments/therapies: Chinese traditional medicine (TCM), western modern medicine (refers to taking western modern medications excluding TCM and other treatments for cancers), surgery, and radiation/chemotherapy. We also identified two types of medical services utilization: Outpatient care (respondents were asked whether they had any outpatient visit in the past month), and inpatient care (respondents were asked whether had any hospital stay in the past year). Medical expenditures were collected during the interviews, including total expenditure, reimbursement, and out-of-pocket expenditures for the outpatient visit in the previous month and inpatient visit in the previous year. For the analysis of economic-related disparity, annual household consumption expenditure including food, entertainment, education, clothing, heating, traveling, fitness expenditures, taxes, and donations was used as a proxy for household economic status.

Financial protection was measured by CHE. In previous studies, two criteria of CHE were derived: (1) out-of-pocket payments (OOP) over 40% of the household’s capacity to pay, or (2) over 10% of total household expenditure [28,29,30]. In this study, we defined CHE as medical OOP expenditure equaling or exceeding 40% of the household’s capacity to pay [29]. Alongside, the household’s capacity to pay (denominator) was defined as the household’s expenditure on non-food consumption, and the OOP expenditure (numerator) was defined as the sum of respondents and their spouses’ medical OOP expenditure on outpatient and inpatient care in the past year. CHE was coded as “yes” if the proportion over 40% and “no” if not. The socioeconomic characteristics, including age, gender, marital status, education level, residence location (rural and urban area), region (east, central, west of China), health insurance, quartile of economic status, and complications were included as covariates.

### 2.3. Statistical Analysis

Chi-square tests were used to analyze the sociodemographic differences in treatment types, health service use, and CHE experience among respondents diagnosed with cancer. Concentration Curves (CC) were used to assess economic-related disparities. The farther the CC lies from the equality line (45 degree line), the greater the degree of disparities in healthcare and expenditure [31]. The multivariable logistic regression models were applied to estimate impacts of the cancer treatment on health service use and CHE, after controlling for sociodemographic characteristics. All statistical analyses were conducted using STATA 15.0. *p* values less than 0.05 were considered as statistically significant.

## 3. Results

### 3.1. Demographic Characteristics of the Study Population

Table 1 reports the descriptive statistics of 381 respondents with identified cancer in 2015. The middle-aged and the elderly with cancer had a higher proportion of women (62.94%) than men (37.06%). In 2015, more than 90% of 381 Chinese adults with cancer were covered by health insurance schemes, most of them enrolling in the New Rural Cooperative Medical Scheme (NCMS) in China. Nearly two-thirds of cancer patients were with at least one or more complications. The per capita household annual consumption expenditure was 21594.8 Yuan (US$ 3237.6). Patients living in urban areas had higher consumption expenditure than those in rural areas.

### 3.2. Cancer Treatment, Health Service Utilization, and Catastrophic Health Expenditure

Table 2 shows the treatment status, health service utilization, and the incidence of CHE among patients with cancer in China in 2015. Overall, over 49% of cancer patients chose taking medicine as the main type of treatment (18.37% TCM and 30.71% western modern medicine), 26.5% received surgery therapy, and 17.59% received radiation/chemotherapy. Compared to patients in rural areas, more urban patients took TCM (14.69% vs. 22.94%). Among all participants, 30.18% used outpatient services, 29.66% used inpatient services, and 26.77% reported CHE. The rural patients suffered a higher incidence of CHE (32.23% vs. 20.00%) than urban individuals. Furthermore, considerable variations were found among medication treatment, non-medication therapy, health service use, and CHE across social-economic subgroups (Supplementary Materials Tables S1 and S2).

### 3.3. Economic-Related Disparities in Healthcare for Cancer

Table 3 revealed the gaps in cancer care use across household economic levels. The outpatient and inpatient services used by patients in the highest economic group were 1.78 and 2.62 times higher than people in the lowest economic group, respectively. The patients with highest economic status had 4.15 times more inpatient visits than people with lowest economic level in urban areas. The wealthiest group also received more than twice the surgery therapy and radiation or chemotherapy of the poorest group.

**Figure 1:**
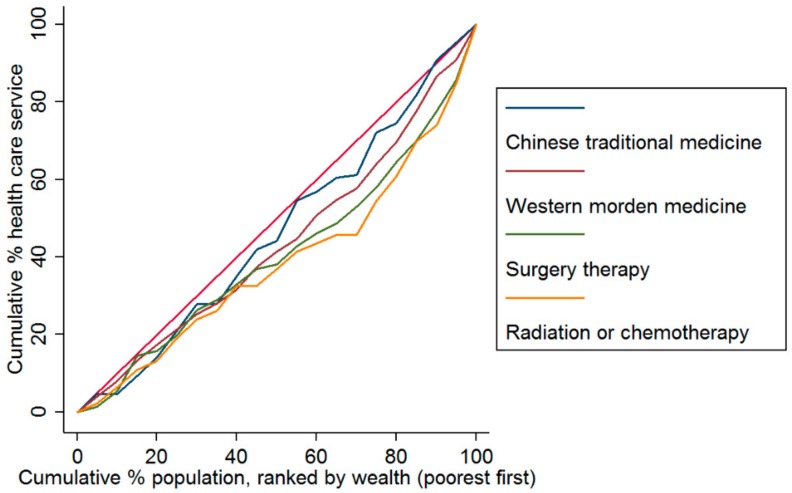
Concentration curves of treatments for patients with cancer in China in 2015.

**Figure 2:**
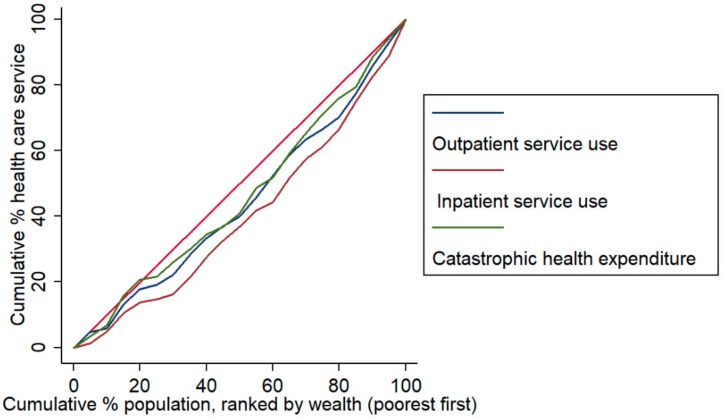
Concentration curves of health service use and expenditure for patients with cancer in China in 2015.

Figure 1 and Figure 2 show the economic-related disparities in treatments, health service use, and CHE among cancer patients. Concentration curves suggested that all indicators and service utilization were more concentrated among the patients with a high economic level than those individuals with a low economic level in 2015. For cancer treatment, the least inequitable indicator was taking TCM (Figure 1). We also found that the economic-related disparity in inpatient service use was higher, compared with the outpatient service (Figure 2).

### 3.4. Impacts of the Cancer Treatment on Health Service Use and CHE

Table 4 reveals a positive association between the treatment of cancer and outpatient visit (Adjusted Odds Ratio (OR) = 2.492, 95% CI = 1.506, 4.125), inpatient visit (AOR = 1.817, 95% CI = 1.098, 3.007), as well as CHE (AOR = 2.744, 95% CI = 1.578, 4.772). In the urban area, taking western modern medicine was positively associated with outpatient visits and surgery therapy increased the inpatient care. However, the radiation/chemotherapy was positively associated with inpatient visits in the rural area, after controlling all sociodemographic covariates. All cancer therapies except for medication treatments were associated with a higher incidence of catastrophic expenditure, particularly the impact of surgery therapy on CHE (AOR = 6.05, 95% CI = 3.393, 27.866) in urban areas.

## 4. Discussion

This study found socioeconomic disparities in service utilization, treatment therapy, and catastrophic expenditure among middle-aged and elderly adults with cancer in China. Age, insurance types, and the household wealth status had impacts on health service use and therapy choosing.

### 4.1. Impact of Cancer Treatment on Health Service Use

This study found all cancer treatments increased hospital service utilization. The outpatient service use increased with medication therapy, and inpatient service use increased with surgery therapy and radiation/chemotherapy. Previous studies also pointed out that cancer treatment played a pivotal role in activating health service utilization [32,33]. Chumbler and colleagues illustrated that living with cancer was associated with an increased frequency of visiting doctors and using hospitalization [32]. Chumbler explained that cancer treatment induced symptoms/complications such as body pain, nausea, fatigue, and mental disorders, therefore patients prefer to visit doctors frequently in outpatient sectors to manage the symptoms. Correspondingly, inpatient service use increased when those outpatients were referred to ward [32]. Additionally, clinical research regarding cancer patients’ self-report symptoms has also indicated that routine cancer treatment resulted in symptoms that precipitated emergency room visits and hospital admissions [33].

### 4.2. Association between Cancer Treatment and CHE

This study suggested that cancer treatment was associated with CHE for cancer patients. Patients receiving surgery therapy and radiation or chemotherapy had a higher risk of experiencing CHE. This finding was consistent with many previous studies [34,35,36]. Huang et al. surveyed 37 tertiary hospitals across 13 Chinese provinces between 2012 and 2014 and concluded that the expenditures of cancer diagnosis and treatment were catastrophic for most patients and varied over types and stages of cancer [34]. Similarly, Choi et al. in South Korea found that approximately 40% of families with cancer patients were exposed to excessive expenditure due to cancer treatment and that the longer the treatment, the higher the family financial burden [35]. Furthermore, a population-based analysis conducted in the United States illustrated that cancer survivors commonly experienced CHE and that for individuals aged between 18 and 64, the economic challenge concerning cancer treatment was particularly tremendous because their employment abilities were limited by health conditions [36].

The reasons why cancer treatment induced CHE could be explained from three aspects. First, medical expenditures on cancer involve multiple aspects ranging from physical examinations, diagnoses to treatment, and subsequent care [37]. The direct expenditures on treating cancer and managing cancer recurrences and comorbidities are high and hard to estimate [38]. As it was estimated in the cross-sectional study with 14,594 cancer patients, the annual income per household was $8607 in 2014 while the average cost per patient spent on cancer was $9739, exceeding the annual household income by about $1000 [34]. Meanwhile, the indirect cost was high as well. Indirect costs were the commencing expenses on managing cancer recurrences and comorbidities as well as the expenses on taking care of the patients [39], and most indirect costs are paid out-of-pocket [40]. Although it was rarely identified, the time cost of caregivers was also a crucial part of cancer expenditures. Yabroff and Kim analyzed a national survey data estimating the time spent on informal care for patients with bladder, breast, and colorectal cancer, or Non-Hodgkins lymphoma (NHL) and concluded that caregivers provided care for those patients up to 8.3 h per day on average over the two years after diagnosis [41]. Summing up the direct and indirect expenditures on cancer treatment, households with cancer patients were undertaking catastrophic financial burden.

Second, cancer could deprive patients of their working abilities, causing the loss of a job and income [42]. A meta-analysis has suggested that people with cancer are 1.37 times more likely to experience unemployment compared to healthy people [42]. With impaired health conditions and reduced employment opportunities, families with cancer patients were vulnerable when confronting excessive medical expense [43].

Third, the occurrence of CHE was associated with the design and protection capacity of insurance schemes [44]. In China, the service coverage and reimbursement rate of three essential health insurance schemes are not sufficient for patients with cancer. It was common before 2015 that people covered by the essential health insurance still needed to pay out-of-pocket for the drugs or therapies not included in the insurance coverage list [45]. In 2018, China’s National Health Insurance Bureau announced that 17 anti-cancer drugs were added to the health insurance reimbursement list. Of the 17 drugs, 12 of them were used to treat solid tumors and five of them were used to treat hematological tumors. These drugs were targeted at cancers such as non-small cell lung cancer, kidney cancer, colorectal cancer, melanoma, and lymphoma which were cancers with high incidence and prevalence in China. Expending the reimbursement list to include the 17 drugs caused a 56.7% reduction of the out-of-pocket cost shared by cancer patients [46]. This study involved cancer patients in 2015 when the reimbursement list was not expended, suggesting a risk of CHE among people who were paying for the 17 drugs by themselves.

### 4.3. Regional and Economic Disparities in Healthcare and CHE

This study found that patients from higher-income families use more advanced treatments, more inpatient services, but experience less CHE than those from lower-income families. Similar disparities were also found among rural and urban residents. This trend could be explained by the association of regional economic development and health resource allocation.

First, the urban–rural partition leads to uneven income levels between urban and rural residents. China’s National Bureau of Statistics calculated that the disposable income of urban residents in 2015 was 31,195 CNY per capita, which was 3 times higher than that of rural residents (11,422 CNY per capita) [47]. Thus, once being exposed to excess medical expenditures on cancer treatment, rural patients are exposed to a higher risk of impoverishment [47].

Second, large gaps exist in health resources allocation between urban and rural areas in China, and rural residents are less likely to receive services of the same quality [48]. China’s National Cancer Centre showed that the top prevalent cancers in rural China were of the digestive system that could be controlled if make an early diagnosis. However, the rural residents face more difficulty in accessing early detection than urban residents [49]. The disparity in the 5 year survival rates among rural patients and urban patients were found [50].

Health insurance schemes also contribute to urban–rural gaps. The essential medical insurance schemes, NCMS, UEBMI, and URBMI were designed based on Hukou and employment status. To be specific, NCMS is designed to enroll rural populations at low premiums and lower benefits, while UEBMI and URBMI are designed to enroll the urban employees and urban residents at higher premiums and better benefits [51]. Though the NCMS and URBMI were consolidated after 2012, gaps in benefit packages still existed among rural–urban employment choices [52]. Meanwhile, NCMS and URBMI are partly financed by county-level governments, so the local economic development is influential [45].

### 4.4. Policy Implications

To bridge the gaps among cancer patients of different socioeconomic characteristics, measures should be taken to improve health insurance protection capacity and bridge health inequities between urban and rural populations.

The range of health insurance coverage regarding cancer treatment should be expended, especially for rural residents enrolled in NCMS. For cancer treatment, the least inequitable indicator was taking Chinese or western medication, compared with radiation, chemotherapy, and surgery. On the one hand, medication treatment is the most common and necessary therapy, especially for the early stage of cancer. On the other hand, the national essential medicine system (EMS) has been developed across the country, which aims to improve medication access, quality, and appropriate use for most NCDs including cancers. Based on the EMS, primary care facilities in townships and counties in China have access to more than 400 essential medicines [53]. Provision of these medicines is heavily subsidized by the China government, with the implementation of zero-profit mark-up for essential medicines. The previously exploited 15% price markup was disallowed [54]. With the development of the EMS and more covered medications in China, the EMS might reduce the economic burden of cancer patients to some extent. Following the health system reform, China achieved a significant increase in insurance coverage, with 95.7% of the Chinese population being covered by public health insurance schemes in 2011 (i.e., Urban Employee Basic Medical Insurance (UEBMI), the Urban Resident Basic Medical Insurance (URBMI), and the New Rural Cooperative Medical Scheme (NCMS)) [5]. Some studies found that social economic development and health insurance determined the level of financial protection among people with cancers [4]. However, the benefits packages and degree of financial protection for patients with NCDs vary across three insurance schemes [6]. The NCMS enrolled by almost rural residents in China covered fewer health service and was still with a low level of financial protection than the UEBMI [55]. Therefore, it is still necessary to further provide financial risk protection and improve the effective coverage and affordability of cancer treatment and health service utilization in China.

Moderating the disparity of health resource allocation between rural and urban China should be considered. Since multiple studies have proved that screening is a cost-effective way to reduce the disease burden of cancer, public health services such as cancer detection, physical examinations, and regular follow-ups were proposed to be promoted and generalized in rural China [56]. Since 2009, the National Public Health Initiative has been performed in China, aiming at promoting healthcare equity and strengthening primary public health services. However, improvements should be made with regard to cancer screening and individual case management. We expect that cancer could be diagnosed and treated at an early stage so that the health outcome would be improved and possibilities of occurring unexpected medical expenditures would be reduced. Last but not least, as impoverishment induced by CHE is a rising concern in China, policies aiming at eliminating poverty should be followed up.

### 4.5. Strengths and Limitations

For the strengthens of this study, we used a nationally representative survey data to examine the socioeconomic disparities and the effect of cancer treatments on outpatient and inpatient visits and CHE in China. This research will contribute to a deeper understanding of the difference in health service use and financial risk influenced by sorts of cancer treatment in China. However, there are several limitations. Firstly, the use of self-reported measures of cancer and health service utilization may underestimate their prevalence, particularly for older persons and those from lower socioeconomic and educational backgrounds who may be more likely to under-report [57]. Secondly, this study only considered the middle-aged and elderly populations in China. The disparity of healthcare for cancer and its impacts among younger populations should be considered in future studies. Thirdly, few participants covered by insurance limits estimates of the effectiveness of health insurance on service use and CHE, although we have compared differences in healthcare for cancers between insurance groups in China. Fourthly, there are around 20% of patients with missing values regarding health expenditures during the survey conducted in 2015. To account for nonresponse bias, we adjusted the analysis by using the created weights for individuals. Last but not the least, the medical information about cancer patients, especially the severity and the stage of the cancer, was not collected in the CHARLS database. There is a limitation for this study to adjust the confounding factors of medical information for cancer patients in the analysis.

## 5. Conclusions

In China, socioeconomic disparities in treatment and health service utilization among middle-aged and elderly cancer patients was largely determined by patient financial capability. The current insurance schemes are insufficient to address these disparities. A comprehensive health insurance policy of expanding the current benefits packages and strengthening the Public Medical Assistance System, are essential for Chinese adults with cancer.

## Figures and Tables

**Table 1:** Demographic characteristics of the study population.

Characteristics	Number	Unweighted Proportion, %	Weighted Proportion, %
Total	381	100.00	100.00
Age (years)			
45–60	185	48.56	50.94
≥60	196	51.44	49.06
Gender			
Male	120	31.50	37.06
Female	261	68.50	62.94
Marital status			
Married/partnered	336	88.19	86.65
Never married/divorced	45	11.81	13.35
Level of education			
Primary school/below	269	70.60	69.27
Middle school/above	112	29.40	30.73
Residence location			
Urban area	170	44.62	56.90
Rural area	211	55.38	43.10
Region			
East	158	41.47	52.11
Central	145	38.06	31.28
West	78	20.47	16.61
Health insurance			
None	41	10.76	9.37
NCMS	218	57.22	43.98
URBMI/others	59	15.49	25.15
UEBMI	63	16.54	21.50
Complication			
None	134	35.17	31.00
1–2	135	35.43	45.06
3 and above	112	29.40	23.94
PCE, Mean (CNY)			
All	259	21,450.27	21,594.80
Rural	150	18,161.54	17,866.11
Urban	109	25,976.04	25,452.49

Note: UEBMI, Urban Employee Basic Medical Insurance; URBMI, Urban Resident Basic Medical Insurance; NCMS, New Rural Cooperative Medical Scheme; Others, government healthcare, private medical insurance and so on; PCE, Per capita household annual consumption expenditure. There are 122 participants with missing value of PCE; CNY, Chinese Yuan.

**Table 2:** The treatment status among Chinese adults with cancer in 2015, by residence location.

Variables	Total		Urban		Rural		*p* Value
	*n*	%	*n*	%	*n*	%	
All patients	381	100.00	170	100.00	211	100.00	
Overall treatments	189	49.61	95	55.88	94	44.55	0.028
Chinese traditional medicine (TCM)	70	18.37	39	22.94	31	14.69	0.039
Western modern medicine (WMM)	117	30.71	58	34.12	59	27.96	0.195
Medication treatment (TCM or WMM)	137	35.96	71	41.76	66	31.28	0.034
Surgery therapy	101	26.51	51	30.00	50	23.7	0.166
Radiation/chemotherapy	67	17.59	32	18.82	35	16.59	0.569
Multiple treatments					0		
TCM & WMM	50	13.12	26	15.29	24	11.37	0.26
Medication & Surgery therapy	61	16.01	31	18.24	30	14.22	0.288
Medication & Radiation/chemotherapy	45	11.81	23	13.53	22	10.43	0.351
Surgery & Radiation/chemotherapy	39	10.24	22	12.94	17	8.06	0.118
Health service use			0		1		
Outpatient visit	115	30.18	46	27.06	69	32.7	0.233
Inpatient visit	113	29.66	55	32.35	58	27.49	0.301
Outpatient & Inpatient visit	44	11.55	18	10.59	26	12.32	0.599
Catastrophic health expenditure	102	26.77	34	20.00	68	32.23	0.007

Note: Overall treatments, refers to the patient has at least one type of treatment/therapy included in this study. TCM, Chinese traditional medicine; WMM, Western modern medicine. *p* value, Chi-square test.

**Table 3:** Healthcare services and catastrophic health expenditure for cancer patients in China across wealth quarters, 2015 (%).

PCE, Quartile	Chinese Traditional Medicine	Western Modern Medicine	Surgery Therapy	Radiation/Chemotherapy	Outpatient Service Use	Inpatient Service Use	Catastrophic Health Expenditure
All							
1st (Lowest)	13.85	24.62	23.08	13.85	24.62	18.46	29.23
2nd	15.38	23.08	21.54	12.31	27.69	27.69	26.15
3rd	18.46	26.15	23.08	12.31	33.85	29.23	40.00
4th (Highest)	18.75	42.19	50.00	32.81	43.75	48.44	39.06
Ratio (highest/lowest)	1.35	1.71	2.17	2.37	1.78	2.62	1.34
*p* value	0.849	0.061	0.001	0.004	0.099	0.002	0.239
Urban							
1st (Lowest)	21.43	28.57	25.00	17.86	21.43	14.29	25.00
2nd	18.52	29.63	18.52	3.70	33.33	22.22	14.81
3rd	22.22	33.33	29.63	18.52	18.52	33.33	25.93
4th (Highest)	25.93	40.74	59.26	37.04	44.44	59.26	37.04
Ratio (highest/lowest)	1.21	1.43	2.37	2.07	2.07	4.15	1.48
*p* value	0.932	0.772	0.008	0.021	0.136	0.002	0.321
Rural							
1st (Lowest)	7.89	18.42	21.05	7.89	28.95	23.68	36.84
2nd	16.22	24.32	27.03	18.92	29.73	21.62	24.32
3rd	10.53	18.42	18.42	10.53	36.84	36.84	52.63
4th (Highest)	16.22	43.24	40.54	29.73	43.24	37.84	43.24
Ratio (highest/lowest)	2.06	2.35	1.93	3.77	1.49	1.60	1.17
*p* value	0.619	0.045	0.134	0.050	0.526	0.274	0.084

Note: PCE, Per capita household annual consumption expenditure. *p* value, Chi-square test.

**Table 4:** Multivariable regression analysis of impacts of the cancer treatment on health service use and catastrophic health expenditure.

Treatment Type	Outpatient Visit			Inpatient Visit			Catastrophic Health Expenditure		
	AOR	95% CI		AOR	95% CI		AOR	95% CI	
**All**									
Chinese traditional medicine	2.156 *	1.193	3.896	1.091	0.589	2.019	1.873	0.987	3.554
Western modern medicine	2.171 **	1.292	3.649	1.124	0.658	1.919	1.358	0.779	2.367
Surgery therapy	2.224 **	1.299	3.809	2.244 **	1.313	3.834	3.697 ***	2.052	6.662
Radiation/chemotherapy	1.373	0.731	2.579	2.504 **	1.369	4.579	2.361 **	1.232	4.526
Treatment overall	2.492 ***	1.506	4.125	1.817 *	1.098	3.007	2.744 ***	1.578	4.772
**Urban**									
Chinese traditional medicine	1.973	0.828	4.697	1.161	0.481	2.805	2.763 *	1.045	7.307
Western modern medicine	2.606 *	1.135	5.981	1.500	0.673	3.343	2.389	0.977	5.839
Surgery therapy	2.077	0.905	4.765	3.487 **	1.509	8.058	9.723 ***	3.393	27.866
Radiation/chemotherapy	2.255	0.852	5.963	2.290	0.889	5.901	3.080 *	1.066	8.898
Treatment overall	2.168	0.968	4.855	2.441 *	1.108	5.379	7.277 **	2.325	22.774
**Rural**									
Chinese traditional medicine	2.315	0.968	5.539	0.906	0.345	2.382	1.303	0.492	3.447
Western modern medicine	1.991	0.975	4.067	0.950	0.436	2.070	1.088	0.500	2.366
Surgery therapy	3.169 **	1.440	6.974	1.802	0.820	3.961	2.726 *	1.212	6.131
Radiation/chemotherapy	1.101	0.457	2.655	3.603 **	1.496	8.682	3.402 *	1.270	9.115
Treatment overall	3.017 **	1.536	5.929	1.654	0.822	3.329	2.133 *	1.057	4.306

Note: The control variables in logistic regressions included: age, gender, marital status, education, residence location, region, health insurance, economic status and complication. AOR, Adjusted Odds Ratio. * *p* < 0.05, ** *p* < 0.01, *** *p* < 0.001, significance test.

## Supplementary Materials

The following are available online at https://www.mdpi.com/1660-4601/17/4/1327/s1, Figure S1a: Concentration curves of treatments for Chinese patients in urban areas in 2015, Figure S1b: Concentration curves of health service use and expenditure for Chinese patients in urban areas in 2015, Figure S2a: Concentration curves of treatments for Chinese patients in rural areas in 2015, Figure S2b: Concentration curves of health service use and expenditure for Chinese patients in rural areas in 2015, Table S1: The treatment status among Chinese adults with cancer in 2015, by sociodemographic groups, Table S2: Health service utilization and catastrophic health expenditure among Chinese adults with cancer, 2015.
