# Supplementary material for: Socioeconomic Disparities in Cancer Treatment, Service Utilization and Catastrophic Health Expenditure in China: A Cross-Sectional Analysis
Source: Int J Environ Res Public Health. 2020 Feb 19;17(4):1327. doi: 10.3390/ijerph17041327 (PMC7068279; doi:10.3390/ijerph17041327)
Supplement: Supplementary file 1 [file ijerph-17-01327-s001.pdf]

International Journal of
Environmental Research and Public Health

MDPI

Supplementary File

Table S1. *The treatment status among Chinese adults with cancer in 2015, by sociodemographic groups.*

Variables	Chinese traditional medicine		Western modern medicine		Surgery therapy		Radiation or chemotherapy	
	%	P value	%	P value	%	P value	%	P value
Age (years)								
45-60	18.38	0.998	31.89	0.627	28.65	0.358	15.68	0.341
>=60	18.37		29.59		24.49		19.39	
Gender								
Male	23.33	0.090	37.50	0.051	32.50	0.072	25.83	0.004
Female	16.09		27.59		23.75		13.79	
Marital status								
Married/partnered	18.45	0.913	31.85	0.189	26.49	0.980	17.86	0.703
Never married/divorced	17.78		22.22		26.67		15.56	
Level of education								
Primary school/below	18.22	0.902	31.60	0.560	24.16	0.108	18.22	0.616
Middle school/above	18.75		28.57		32.14		16.07	
Region								
East	17.09	0.670	25.95	0.061	25.95	0.438	18.99	0.158
Central	17.93		30.34		29.66		20.00	
West	21.79		41.03		21.79		10.26	
Health insurance								
None	14.63	0.029	29.27	0.050	24.39	0.206	19.51	0.189
NCMS	14.22		25.69		23.39		14.22	
URBMI/others	27.12		38.98		28.81		20.34	
UEBMI	26.98		41.27		36.51		25.40	
Complication								
None	14.93	0.212	32.84	0.296	23.88	0.019	17.91	0.017
1-2	22.96		33.33		34.81		23.70	
3 a d above	16.96		25.00		19.64		9.82	
Total	18.37	-	30.71	-	26.51	-	17.59	-

Note: UEBMI, Urban Employee Basic Medical Insurance; URBMI, Urban Resident Basic Medical Insurance; NCMS, New Rural Cooperative Medical Scheme; Others, government healthcare, private medical insurance and so on; PCE, Per capita household annual consumption expenditure.

Table S2. Health service utilization and catastrophic health expenditure among Chinese adults with cancer, 2015.

Variables	Outpatient visit		Inpatient visit		Catastrophic health expenditure	
	%	P value	%	P value	%	P value
Age (years)						
45-60	30.39	0.725	23.50	0.008	28.66	0.110
>=60	32.09		36.08		37.16	
Gender						
Male	28.70	0.476	38.66	0.012	37.23	0.261
Female	32.41		25.97		30.73	
Marital status						
Married/partnered	30.25	0.260	30.12	0.866	32.86	0.842
Never married/divorced	38.64		28.89		31.03	
Level of education						
Primary school/below	31.27	0.988	31.20	0.420	35.16	0.154
Middle school/above	31.19		27.03		26.88	
Region						
East	31.82	0.479	28.03	0.222	26.56	0.133
Central	28.06		34.97		35.54	
West	36.00		24.68		39.68	
Health insurance						
None	39.47	0.124	17.95	0.010	27.27	0.054
NCMS	34.27		30.41		37.50	
URBMI/others	22.41		20.69		34.62	
UEBMI	23.73		44.44		17.65	
Complication						
None	28.24	0.024	25.95	0.275	30.77	0.529
1-2	25.95		34.81		30.77	
3 and above	41.51		28.83		37.36	
Total	31.25	-	29.97	-	32.69	

Note: UEBMI, Urban Employee Basic Medical Insurance; URBMI, Urban Resident Basic Medical Insurance; NCMS, New Rural Cooperative Medical Scheme; Others, government healthcare, private medical insurance and so on; PCE, Per capita household annual consumption expenditure.

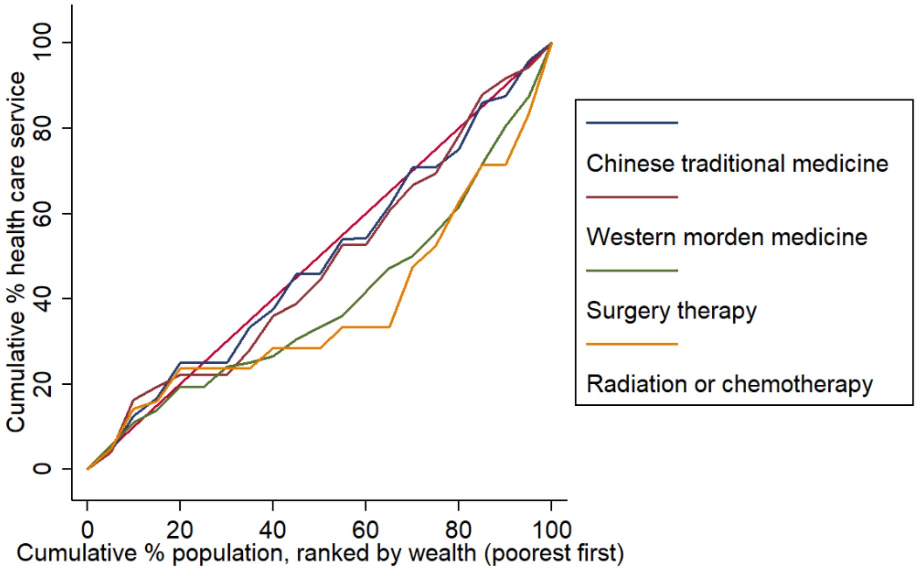

Figure S1a. *Concentration curves of treatments for Chinese patients in urban areas in 2015.*

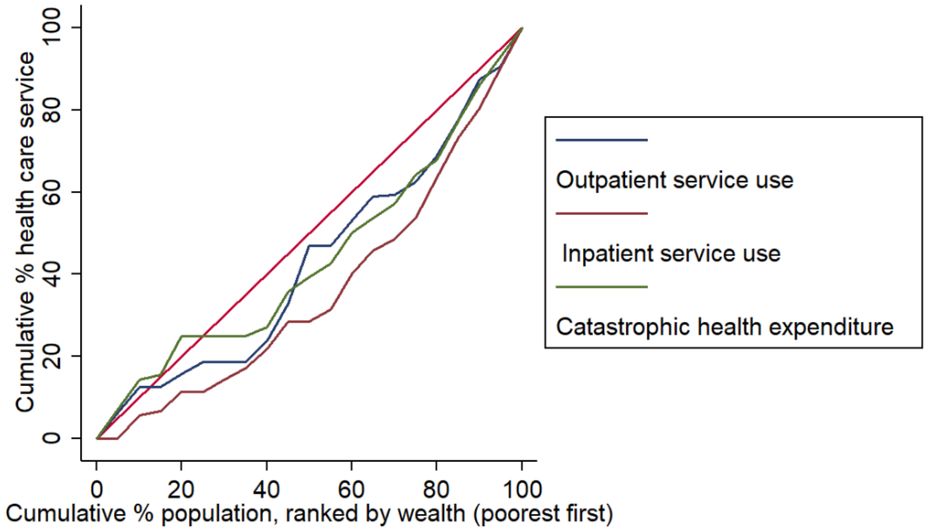

Figure S1b. *Concentration curves of health service use and expenditure for Chinese patients in urban areas in 2015.*

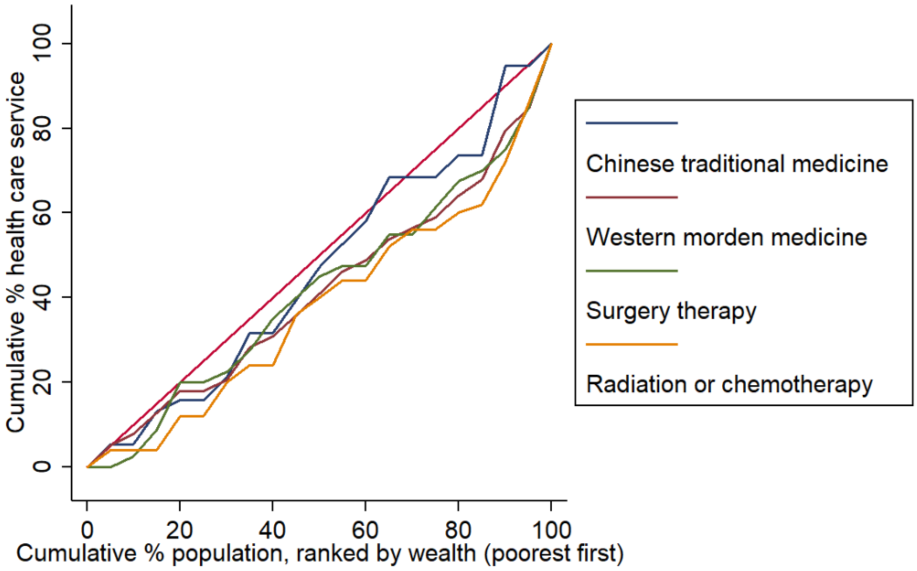

Figure S2a. *Concentration curves of treatments for Chinese patients in rural areas in 2015.*

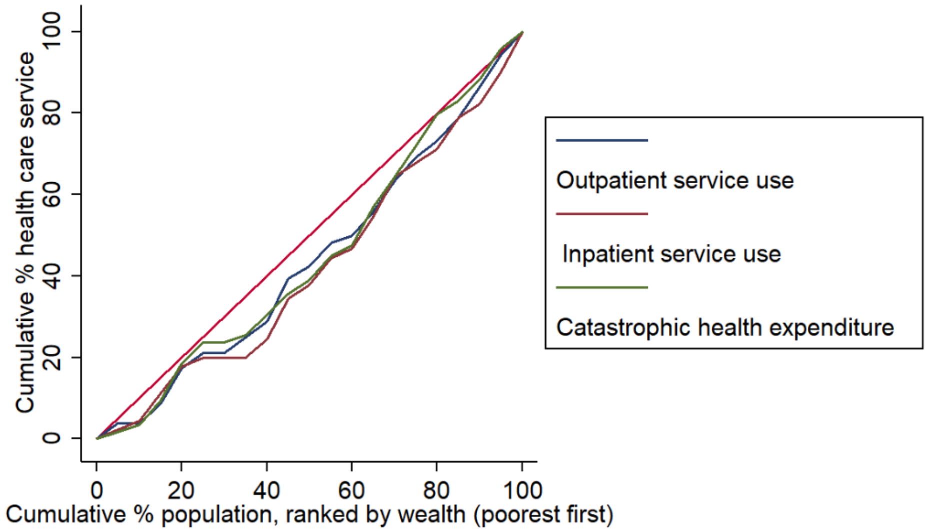

Figure S2b. *Concentration curves of health service use and expenditure for Chinese patients in rural areas in 2015.*
